# Supplementary material for: Complexome profiling of the Chlamydomonas psb28 mutant reveals TEF5 as an early PSII assembly factor
Source: Plant Cell. 2025 Mar 17;37(6):koaf055. doi: 10.1093/plcell/koaf055 (PMC12164590; doi:10.1093/plcell/koaf055)
Supplement: koaf055_Supplementary_Data [file koaf055_supplementary_data.zip › psb28_interactive.zip!/plots/intensity/qualityPlots/peptides/_Cre11.g477700.t1.1.html]

### Cre11.g477700.t1.1

Trivial name:   
SeqCoverage [median]: 1.20  
Mass: 95.00 kDa  
  
 

| Sequence | MissedCleavage | Mass [Da] | wtIntensity | muIntensity | Score |
| --- | --- | --- | --- | --- | --- |
| VLRLNCMDLNR | 1 | 1345.70 | 44917.16 | 7938.50 | 113.71 |
|  |  |  | 44917.2 | 7938.5 |  |
